# Efficacy of Artemisinin Base Combination Therapy and Genetic Diversity of *Plasmodium falciparum* from Uncomplicated Malaria *Falciparum* Patient in District of Pesawaran, Province of Lampung, Indonesia

**Published:** 2019

**Authors:** Jhons Fatriyadi SUWANDI, Widya ASMARA, Hari KUSNANTO, Din SYAFRUDDIN, Supargiyono SUPARGIYONO

**Affiliations:** 1. Department of Microbiology and Parasitology, Faculty of Medicine, University of Lampung, Bandar Lampung, Indonesia; 2. Department of Microbiology, Faculty of Veterinary Medicine, Gadjah Mada University, Jogjakarta, Indonesia; 3. Department of Public Health, Faculty of Medicine, Public Health and Nursing, Gadjah Mada University, Jogjakarta, Indonesia; 4. Department of Parasitology, Faculty of Medicine, Hasanuddin University, Makasar, Indonesia; 5. Eijkman Institute for Molecular Biology, Jakarta, Indonesia; 6. Department of Parasitology, Faculty of Medicine, Public Health and Nursing, Gadjah Mada University, Jogjakarta, Indonesia

**Keywords:** Artemisinin combination therapy, Efficacy, Genetic variation, *Plasmodium falciparum*

## Abstract

**Background:**

Malaria is an infectious disease caused by *Plasmodium* sp., that still prevalence in some part of Indonesia. District of Pesawaran is one of malaria endemic area in the Province of Lampung. The purpose of this study was to evaluate the efficacy of the ACT treatment in the District of Pesawaran Province of Lampung, Indonesia from Dec 2012 to Jul 2013 and the genetic variation of the *Plasmodium falciparum* also studied.

**Methods:**

This study was observational analytic study of falciparum malaria patients treated with ACT and primaquine (DHP-PQ and AAQ-PQ) at Hanura Primary Health Centre (*Puskesmas*). DNA isolation was done with QIAmp DNA Mini Kit. Amplification of *PfMDR1*, *MSP1*, and MSP2 genes was done with appropriate forward and reverse primer and procedures optimized first. PCR Product of PfMDR1 gene was prepared for sequencing. Data analysis was done with MEGA 6 software.

**Results:**

The results of this research are DHP-PQ effectiveness was still wellness among falciparum malaria patients in District of Pesawaran, Province of Lampung, Indonesia. There is Single-nucleotide mutation of *N86Y* of *PfMDR1* gene. The dominant alleles found are MAD20 and 3D7 alleles with Multiplicity of Infection (MOI) are low.

**Conclusion:**

Therapy of DHP-PQ as an antimalarial falciparum in Pesawaran District, Lampung, Indonesia is still good. The genetic variation found was the SNP on the N86Y PfMDR1 gene, with dominant allele MAD20 and 3D7.

## Introduction

Malaria infection is one of tropical infectious disease in Indonesia. *Plasmodium falciparum* is one of the most predominant causes of malaria diseases in the district of Pesawaran, the province of Lampung, in the south part of Sumatera island of Indonesia (1–3). Based on the annual incidence of these diseases, the district of Pesawaran was classified into medium case incidence area, with Annual Parasite Incidence ranged between 1%–5% population (1,4). Efforts to control this diseases have been the high priority of the district as well as the Provincial Government, through early case detection thoroughly prompt treatment and vector management such as Indoor Residual Spraying (IRS), distribution of Long Lasting Insecticide Treated Net (LLIN) and mosquito breeding site management. Besides the total decreased of the parasite prevalence around the recent, however, epidemic outbreak is still to occur in same villages, and antimalaria drug resistance has been reported to be one of important conditions need to be detailed. Plasmodium resistance against antimalarial drugs is one of the factors that can inhibit malaria control. Since 2004, the government of Indonesia had been implemented Artemisinin-based Combination Therapy (ACT) as a standard treatment of malaria throughout the region in Indonesia. In District of Pesawaran, there are two types of drugs combination that used in malaria patients such as Artesunate-Amodiaquine Primaquine (AAQ-PQ) and Dihydroartemisinin Piperaquine Primaquine (DHP-PQ) (1,2,5).

The rapid clearance of parasite from the blood circulation at malaria patients treated with this ACT standard has been noted from all endemic area in Indonesia. However, the efficacy of these ACT standard treating used to be evaluated. The use of ACT since 10 years ago may cause a decrease in the effectiveness of ACT against Plasmodium. Incidence of malaria at District of Pesawaran remains high from year to year (annual parasite incidence (API) value > 1) (4) is one of the reasons for reviewing the effectiveness of these antimalarials. *Plasmodium falciparum* multidrug resistance-1 (*PfMDR1*) gene is one of the transporter genes that play a role in regulating the pH in the food vacuole of Plasmodium. Polymorphisms in *PfMDR1* gene has been shown to play a role in the change of Plasmodium susceptibility against amodiaquine, mefloquine, lumefantrine, halofantrine and artemisinin with different mechanism. Position N86 (wild-type) plays an important role in the increased resistance to arylaminoalcohol quinolines, such as mefloquine, lumefantrine, and halofantrine, while the position 86Y (mutant) plays an important role in the increased resistance to 4-aminoquinoline such as chloroquine and amodiaquine. Codon 86 of *PfMDR1* gene, have an important role of fluids efflux, including drugs, from the parasite food vacuole into the cytoplasm (6–12).

In addition, genetic variation of *Plasmodium* sp. also affects its susceptibility to antimalarials. To investigate genetic variation, *MSP1, MSP2* and *GLURP* genes were examined. These genetic variants are also associated with the nature of *P. falciparum*. On examination of this genetic variation will show K1, MD20 and RO33 alleles based on *MSP1* gene and FC27 and 3D7 alleles based on MSP1 gene. Genetic variation data on *P. falciparum* in Pesawaran District has not been reported. Therefore, it is important to study genetic variation.

The purposes of this study were evaluate the efficacy of the ACT treatment in the District of Pesawaran; evaluate the nucleic acid changes of the *PfMDR1*, and genetic variation of *P. falciparum* will also bee studied.

## Materials and Methods

### Study site

This study was conducted at Pesawaran District, Lampung Province, Indonesia. Hanura public health center (PHC) which has the highest API (API 2010 and 2011 are 14,07 and 45,21 respectively) was selected (4). The location of this health center is coastal area in the southern part of the district.

### Research procedure and design

This research is analytical survey research. *P. falciparum* positive patients were recruited in Hanura Health Centre from Dec 2012 to Jul 2013.

After the informed concern process and signature of informed consent by the eligible subject, finger prick blood sample was collected for blood smear and spotted on Whatman chromatographic paper (3MM) for parasite DNA analysis. After taking blood samples, the research subjects received ACT treatment according to the standard of malaria treatment issued by the Indonesian Ministry of Health (Day 0). A cohort of blood samples collection for evaluation of parasitemia was done on Day 1, 2, 3, 7, 14, 21 and 28 after ACT administration. Evaluation of the treatment responses of each case was evaluated using standard protocol of the WHO guidelines (13,14).

### Blood sample and data collection

The process of selecting research subjects was done by referring to the inclusion criteria according to the WHO protocol in 2009 (13). At the minimum sample calculation, 50 subjects were obtained (15). During the data collection process, there were 71 samples, but only 62 blood samples of malaria patients matched the WHO inclusion criteria so that all blood samples were analyzed. In each subject, microscopic examination was carried out to determine the parasite density. Blood samples were also taken for DNA analysis examination, by dripping blood on what man chromatographic paper (3MM) filter paper. The thin and thick blood slides were Giemsa stained and parasitemia was examined by qualified microscopic from the district health office and checked by microscopic from Department of Parasitology, Faculty of Medicine, University of Lampung. Dry blood spot was stored in −20 °C until DNA processes.

### Deoxyribose Nucleic Acid (DNA) Extraction from Blood Spot Sample

Dry blood spot on Whatman paper was cut and put into 1.5 ml Eppendorf tube and DNA was extracted using QIAmp DNA Mini kit from Qiagen. The DNA extraction was carried out in accordance with the standard protocol provided within the kit (16).

### Amplification of PfMDR Gene

*PfMDR* gene was amplified using publish primer sequences of Humphreys et al. (10). This primer flanking segment of 578 bp containing codon 86 and 184 of *PfMDR1* gene (10). To determine the genetic condition of *PfMDR1* gene fragments, sequencing of PCR products was performed. The sequencing results were analyzed using MEGA 6 software.

### Amplification of MSP1 and MSP2 Genes

Genotyping of Plasmodium falciparum using PCR was performed on the *MSP1* and *MSP2* genes. The amplification procedure refers to the procedure published and also by performing optimization in the laboratory (17). Examination of genetic variation is only done on samples from the subject with failed therapy and some subjects with adequate therapy.

### Ethical approval

The ethical clearance for this study was approved by the Medical and Health Research Ethics Committee (MHREC) Faculty of Medicine, Gadjah Mada University, Yogyakarta, Indonesia and the Medical and Health Research Ethics Committee (MHREC) Faculty of Medicine, University of Lampung, Lampung, Indonesia (number: KE/FK/804/EC. Date of approval: Oct 23, 2012).

## Results

Artemisinin-Base Combination Therapy drugs used in District of Pesawaran for treat of malaria patients were consists of two types such as Dihydroartemisinin Piperaquine (DHP) and Artesunate-Amodiaquine (AAQ). Both of these drugs were combined with Primaquine (PQ) 0.75 mg/kgbw single dose. Table 1 shows the parasitological monitoring of research subjects, which successfully evaluated for 28 d after ACT treatment. Overall, 38 subjects received DHP-PQ and 24 subjects received AAQ-PQ. Table 1 also shows the treatment response according to WHO criteria. Early Treatment Failure is not found in this study. There were found 6 positive *P. falciparum* of D14 on microscopic examination, while the D28 has 2 positive *P. falciparum*. The *P. falciparum* positive at D14 and D28 are categorized as late treatment failure.

**Table 1:** Parasitological monitoring of research subject after treatment

***Total Subject at D0***	***Number of subjects positive after treatment (%)***				
	D3	D7	D14	D21	D28
		DHP-PQ Combination			
38	0 (0.00)	0 (0.00)	0 (0.00)	0 (0.00)	2 (5.26)
		AAQ-PQ Combination			
24	0 (0.00)	0 (0.00)	6 (25.00)^*^	0 (0.00)	4 (16.67)^*^

* found in the same study subjects

Statistical analysis showed a significant association between response therapy with the type of drug administered (*P*=0.024). Adequate Clinical and Parasitological Response (ACPR) are more common in subjects treated with DHP-PQ compared to subjects treated with AAQ-PQ. Combination AAQ-PQ has a high failure rate (25.00%) compared with DHP-PQ (5.26%) (Table 1).

Microscopic examination on Day 0 showed that asexual stage parasite density varied between 1.000 to 111.440 parasites/μL blood. Microscopic examination on the first day after treatment, 73.68% of the study subjects who received DHP-PQ treatment and 95.83% of the study subjects who received AAQ-PQ treatment were not found asexual stadiums. The third day observation has not found any asexual stage in all study subjects. Parasite density tends to decrease from day to day after treatment at day 0. In this study has not found signs of a shift in parasite clearance time even though it has been found late treatment failure (possibility recrudescence).

Molecular analysis of the *PfMDR1* gene performed on 50 blood samples from 62 samples showed a change in nucleotide base sequence in the fifty samples. The nucleotide base of Adenine (A) at position 256 turns into Thymine (T). The nucleotide encoding codon 86, which causes amino acid changes from Asparagine (N) becomes tyrosine (Y). The nucleotide number 551, which encodes codon 184 reported to have undergone a change in previous research, found no changes in this study.

Genetic analysis was continued by genotyping on *MSP1* and *MSP2* genes (17). The results of genotyping based on *MSP1* gene showed MD20 (84, 85%), RO33 (12, 12%) and K1 (3, 03%) alleles, whereas MSP2 gene was found by FC27 (41, 46%) and 3D7 (58, 54%) alleles. Blood samples from subjects that fail therapy, performed genotyping to analysis of multiplicity of infection (MOI). Seven blood samples of eight blood samples of subjects failed therapy successfully performed molecular analysis. Blood samples from failed therapy subjects were taken at D0 and when the parasites reappeared (D14 and D28). Subjects with adequate therapy were examined for genetic variation in 5 blood samples taken at D0 for comparison. Alleles found based on the *MSP1* gene are MD20, RO33, and K1, whereas in the MSP2 gene found 2 alleles that are FC27 and 3D7. Multiplicity of infection (MOI) based on allele is low (1.57 in *MSP1*, and 1.58 in *MSP2*).

## Discussion

Artemisinin-based Combination Therapy in PHC Hanura has been used since 2004 after the Ministry of Health decided ACT as the standard treatment of malaria. Within 10 years after the first use of ACT, it is possible there has been a decrease in effectiveness. According to WHO criteria, antimalarial drugs are not used anymore, if the treatment failure rate ≥ 10% (WHO 2010). Based on this, the effectiveness of the AAQ-PQ has been reduced compared with the DHP-PQ.

Subjects with therapeutic response late parasite failure (LPF) mostly parasites appeared at day 14 (75%) and day 28 (25%). The parasite was not found on examination D 2, 3 and 7 and reappeared on D 14 or 28. These conditions indicate recrudescence has arisen. Recrudescence is a re-invention of the parasite due to persistence of the parasite in the blood, but there is a decrease so it is not detected in microscopic examination. *P. falciparum* takes 24–72 h to develop from the ring stage to mature schizont stage (18). Levels of artemisinin (dihydroartemisinin or artesunate) in the blood will rapidly decline after the third day of treatment (half-time about 1 hour). Treatment with single artemisinin needs less than 7 d or 3 d when combined with other long-acting drugs (19–21). A decrease in the concentration of artemisinin after the third day of treatment will have sufficient time for the parasite remnant to do schizogony, so within 10 d of the parasite can be found back in the peripheral blood.

The decreased of AAQ-PQ effectiveness is probably related to polymorphism at codon N86Y *PfMDR1* genes. Polymorphism at codon N86Y cause amodiaquine is not effective in eliminating parasites. This condition makes the high treatment failure in patients who received AAQ-PQ (AAQ-PQ treatment failure is 24.00%). This is due to an increase in pH and reflux of food vacuole so amodiaquine concentration in the food vacuole will decrease. High pH of the food vacuole will influence the amodiaquine to inhibit polymerization hem. The results of this study are consistent with another research which states that a single mutation of codon N86Y can cause changes in the function of the transport of fluids and drugs (amodiaquine) significantly (8). In this study, amodiaquine has failed to perform the role as a combination of long-acting drug in the treatment of malaria so that it causes a high failure on AAQ-PQ (> 10%).

The *PfMDR1* gene polymorphism has been shown to play a role in the change of Plasmodium susceptibility to antimalarial amodiaquine, mefloquine, lumefantrine, halofantrine and artemisinin with different mechanisms. Position N86 (wild-type) plays an important role in the increased resistance to arilaminoalkohol quinolines, such as mefloquine, lumefantrine, and halofantrine, while the position 86Y (mutant) plays an important role in the increased resistance to 4-aminoquinoline such as chloroquine and amodiaquine (6–12). The use of ACT in 10 years is not easily changing the nucleotides in the gene *PfMDR1*, especially at codon 86 and 184. In Hanura, Codon 86 of *PfMDR1* gene has been mutated since before the ACT use, whereas codon 184 still wild type (22). The results are consistent with research conducted and found a decrease in the effectiveness of amodiaquine in subjects with falciparum malaria in West Sumba, East Nusa Tenggara, although the treatment failure rate still small (<10.00%) (23).

Different conditions found in subjects who received therapy DHP-PQ, which has a small failure rate (4.55%). Piperaquine as artemisinin combination still gives a good effect as a long-acting drug. In short, a half-time of artemisinin causes the concentration of drug in the body rapidly declined after the third day of treatment. The role of parasite elimination will be taken over by piperaquine. This can be seen in subjects who received treatment with DHP-PQ, still give good therapeutic results with failure rate 4.55%, although it has been found polymorphisms in codon N86Y, *PfMDR* genes from all blood samples subject. Possible mechanisms of resistance to piperaquine are not related to polymorphism at codon N86Y *PfMDR1* gene (24), so the effectiveness of DHP-PQ still optimal, when compared with the AAQ-PQ.

Based on the genotyping results on the *PfMDR1, MSP1* and *MSP2* genes, the genetic conditions of *P. falciparum* isolate Pesawaran classified as low genetic diversity with dominant alleles were MAD20 based on *MSP1* gene and 3D7 allele based on MSP2 gene. This study is in line with the research in South Sumatra, with the dominant allele result being MAD20 (25). Alele K1 is found but not dominant, while RO33 is not found. The three alleles of the *MSP1* gene were also found in previous studies. In Iran, Laos, Myanmar and several places in Africa, strains of MAD 20, K1 and RO33 were always found, but with different percentages (26–31). In Pakistan (32); Myanmar (27) and Cameroon (33), the dominant MAD20 allele found in South Sumatra and Pesawaran Lampung. This study differs from studies in Laos, Iran, and Congo showing the dominant K1 allele found (26,30). Similarly, studies in Malaysia and Sudan showed that the dominant RO33 allele was found (34,35). The genetic isolates of the offerings may have similar properties with isolates in South Sumatra Indonesia, Pakistan, Myanmar, and Kameron

The low MOI of this Pesawaran isolate shows little genetic diversity. The MOI values of this study were in line with the MOI obtained in the Malaysian study (1.37), Pakistan (1.25) and India (1.38) (28, 32, 34), but in South Sumatra (3.60) (25) different from the results in Pesawaran. This condition can be caused by very little new genetic influx from elsewhere or at least the genetic mutation process in *Plasmodium* isolate Pesawaran. The geographical location of the isolated work area of Hanura Puskesmas and the mobility of the population from the minimal outer area is also one of the causes of the least genetic variation of *Plasmodium* in the region. This level of diversity is influenced by transmission rates, population migration, vector population, environmental conditions, human as host, and parasite susceptibility patterns within a region play a role in determining the genetic structure of the parasitic population (25).

## Conclusion

Therapy of DHP-PQ as an antimalarial *falciparum* in Pesawaran District, Lampung, Indonesia is still good. The genetic variation found was the SNP on the N86Y *PfMDR1* gene, with dominant allele MAD20 and 3D7.
